# Frequency of the acquired resistant mutation T790 M in non-small cell lung cancer patients with active exon 19Del and exon 21 L858R: a systematic review and meta-analysis

**DOI:** 10.1186/s12885-018-4075-5

**Published:** 2018-02-06

**Authors:** Zan-Feng Wang, Sheng-Xiang Ren, Wei Li, Guang-Hui Gao

**Affiliations:** 1 Department of Respiratory Medicine, The First Affiliated Hospital of China Medical University, Shenyang, Liaoning China; 2 Department of Medical Oncology, Shanghai Pulmonary Hospital,Tongji University, Tongji University Medical School Cancer Institute, Shanghai, China

**Keywords:** Non-small cell lung cancer, Epidermal growth factor receptor,T790 M, Re-biopsy, Meta-analysis

## Abstract

**Background:**

Although EGFR-TKI is the preferred treatment for NSCLC patients with sensitive mutations, subsequent drug resistance is almost inevitable. The specific mechanisms of EGFR-TKI drug resistance can be identified through repeat biopsy.

**Methods:**

To better understand the clinical characteristics of TKI resistance in NSCLC patients, we retrospectively reviewed studies of acquired TKI drug resistance using repeat biopsy from the last decade. The relevant literature was retrieved from January 2005 to August 2015 in the databases Medline and Embase. The search terms were NSCLC or non-small cell lung cancer and T790 M.

**Results:**

A total of 478 patients with NSCLC tested by repeated biopsy were confirmed to have acquired TKI resistance. Analysis indicated that 240 patients (50.21%) of the 478 patients with acquired TKI drug resistance had the T790 M mutation. The detection rate of T790 M in different repeat biopsy sites was also different, with the highest positive rate in the lymph nodes (60%) and the lowest detection rate in cerebrospinal fluid (less than 5%). In addition, patients with T790 M had longer overall survival compared to those without the mutation (*P* < 0.05). Of the 240 patients with T790 M mutations, 213 patients showed results consistent with the mutation analysis before TKI treatment, and the rate of patients with the L858R point mutation along with the T790 M mutation was lower than that of patients with the exon 19 deletion (36.42% to 58.30%).

**Conclusions:**

T790 M occurred more frequently in patients with the exon 19 deletion than in those with exon 21 L858R, which gave the survival benefit of the T790 M mutation and may explain why patients with the exon 19 deletion had an improved overall survival.

## Background

Studies over the last decade [1–4] have demonstrated that somatic activating mutations in the tyrosine kinase domain of epidermal growth factor receptor (EGFR), including deletions in exon 19 (del19) and point mutations in exon 21 (L858R), are important mediators of cancer cell oncogenesis, proliferation and survival. Discovery of the EGFR-targeting agents gefitinib and erlotinib has provided significant insights into the biologic behaviors of non-small cell lung cancer (NSCLC). Gefitinib and erlotinib are first-generation EGFR-tyrosine kinase inhibitors (TKIs), and both agents play key roles in the treatment of EGFR-mutated NSCLC. However, the median progression-free survival (PFS) for NSCLC patients treated with gefitinib or erlotinib was only 10–12 months. L858R EGFR mutations in patients process less benefit, indicating that EGFR del19-positive disease may be different from those with L858R-positive [5]. Although the initial response to EGFR-TKIs is similar in NSCLC patients with del19 and point mutations in exon 21 (L858R), PFS and OS are significantly greater in patients with del19 than L858R [6–8]. The reason for this difference is currently unknown. Studies investigating repeat biopsies from patients with NSCLC who acquired resistance to erlotinib or gefitinib have demonstrated that the primary cause of drug resistance is the development of drug resistance mutations. Because these mutations substantially impact disease progression in patients with NSCLC, the prognostic difference between EGFR-TKI-treated patients with del19 and L858R might be attributable to differences in the mechanisms underlying drug resistance.

Data obtained from repeat biopsies revealed that the most common drug resistance mutation in patients with NSCLC is a point mutation in EGFR that results in the substitution of threonine with methionine at amino acid position 790 (T790 M) [9]. However, the sample size was too small to examine differences in outcomes between del19 and exon 21 L858R mutations. We conducted a systematic review of repeat biopsy studies in patients with NSCLC who developed resistance to EGFR-TKIs, so as to determine if there was a difference in the incidence of the T790 M EGFR mutation between patients with deletions in exon 19 and point mutations in exon 21 (L858R). In addition, we investigated the association of the T790 M mutation with clinicopathological features of patients with NSCLC.

## Methods

### Study design and search strategy

We searched the PubMed, Medline and Embase databases for relevant articles published before or on August 2015. We conducted a systematic review of articles published between January 2005 and August 2015 using the Medline and Embase databases using the following search terms: NSCLC and T790 M. We only selected articles published in English. Case studies, letters, reviews and editorials were excluded from the analysis. Articles were required to meet the following criteria for inclusion in the meta-analysis: 1) the patients in the study had histologically verified NSCLC, and these patients were confirmed to have a clear EGFR-TKI-sensitive mutation by Droplet Digital Polymerase Chain Reaction (DDPCR) sequencing and 2) the patients underwent treatment with a first-generation EGFR-TKI (primarily gefitinib and erlotinib) and had undergone a repeat biopsy to test for drug resistance mutations because their disease had progressed despite an initial effective response to therapy.

### Selection of trials

**Fig. 1:**
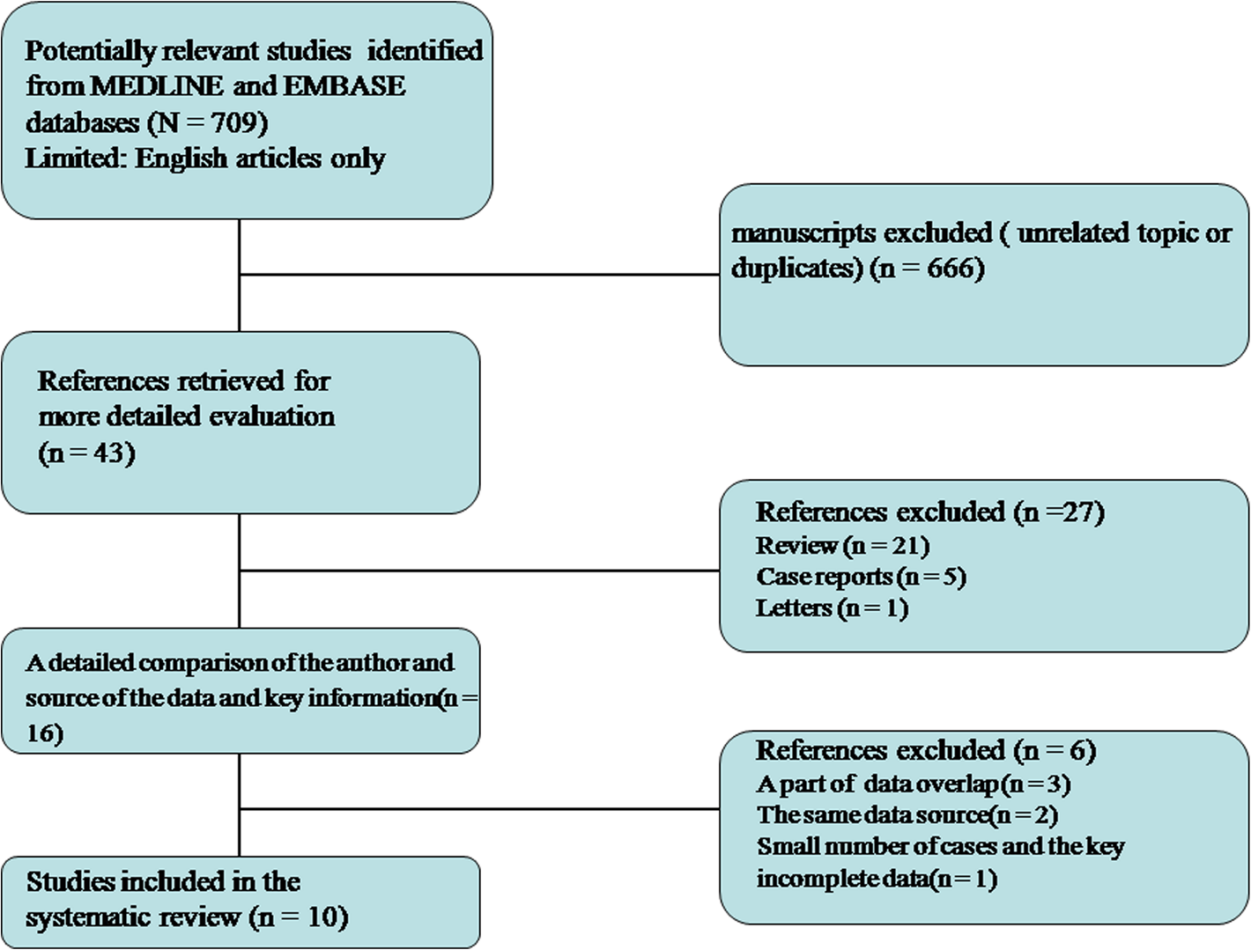
Flowchart of this study

A total of 16 articles met the inclusion criteria and were selected for further analysis (Fig. 1). Data in 4 of the 16 studies originated from the same source (primarily Memorial Sloan-Kettering Cancer Center, Weill Medical College of Cornell University, NY, USA). The article that included the most patients and provided the most information was selected for further analysis to avoid redundancy. Two studies exclusively included patients from the University of Occupational and Environmental Health (Kitakyushu, Japan); therefore, only one of the two studies was retained for analysis. Another study was excluded because it included only 6 patients and did not report EGFR mutation status after the occurrence of drug resistance. A total of 10 clinical studies [10–19] were ultimately included in this systematic review.

### Data extraction and analysis

The 10 studies included a total of 478 patients with NSCLC, pathologically confirmed EGFR-sensitizing mutations, and repeated biopsy to test drug resistance mutations after TKI treatment. The clinical characteristics of patients, including age, sex, smoking status, type of EGFR mutations, repeated biopsy specimens and OS sources, are shown in Table 1. Two reviewers independently assessed the eligibility of the studies identified by the search. We used Stata 11 software to analyze all data and assessed publication bias by Begg’s Test. The heterogeneity was assessed using the Cochran Q test, and statistically significant heterogeneity was defined as *P* < 0.10 and I^2^>50%. We used the random effects model of the Mantel-Haenszel method to calculate hazard ratio (HR) and 95% confidence interval (CI). In addition to the heterogeneity test, the difference was statistically significant at *P* < 0.05. Due to the lack of detailed OS data for all patients, we were unable to perform a median test. Thus, we compared the operating systems between groups after the median was converted to the means as described by Hozo et al. [20]

**Table 1:** Study characteristics

study	No. of patients	Gender M/F	Age (< 70/≥70)	Smoking status (C or F /N)	Treatment	Detecting Items for Drug-Resistance Mechanism
Uramoto H et al. [10]	11	3/8	7/4	2/9	gefitinib	T790 M, KRAS,PTEN MET amp, HGF status
Kuiper JL et al. [11]	66	14/52	NA	30/33^a^	erlotinib gefitinib	T790 M, SCLC, KRAS
Sun JM et al. [12]	70	18/52	NA	14/56	erlotinib gefitinib	T790 M
Li W et al. [13]	54	29/25	49/5	7/47	erlotinib gefitinib Icotinib	T790 M
Sequist LV et al. [14]	37	15/22	31/6	NA	erlotinib gefitinib	T790 M, MET amp, SCLC, PIK3CA, EMT
Kosaka T et al. [15]	14	4/10	NA	6/8	Gefitinib	T790 M, KRAS
Oxnard GR et al. [16]	93	33/60	NA	32/61	erlotinib gefitinib	T790 M, MET amp, HER2
Hata A et al. [17]	78	24/54	51/27	24/54	erlotinib gefitinib	T790 M
Chen HJ et al. [18]	29	18/11	27/2	6/23	erlotinib gefitinib	T790 M, MET amp
Ji W et al. [19]	26	10/16	21/5	NA	gefitinib	T790 M, MET amp, AXL, EMT, CD56

^a^Uncertain for the 3 cases; C or F /N = current or former /never smoker

## Results

**Fig. 2:**
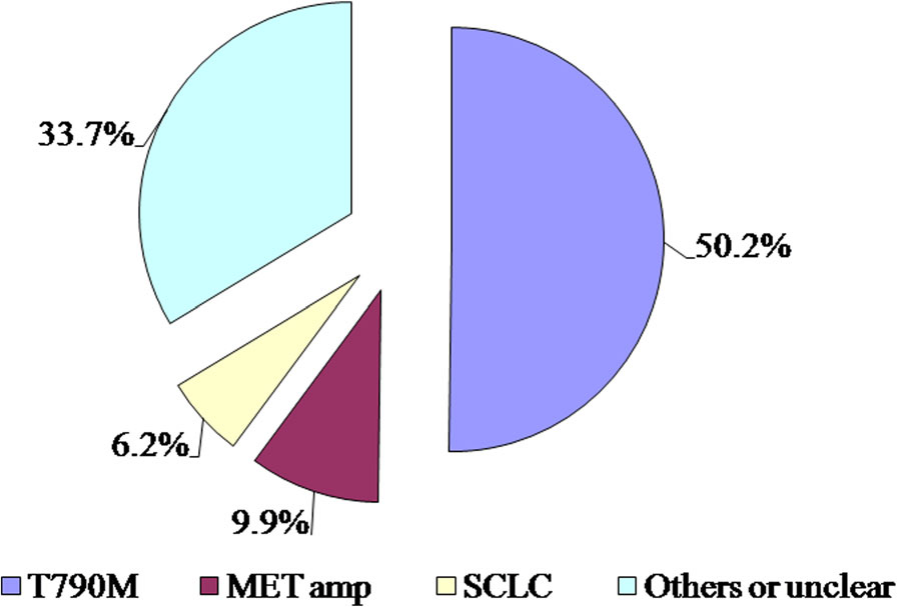
The relative frequencies of the various mechanisms of acquired resistance

We analyzed potential mechanisms mediating drug resistance in 478 patients with NSCLC who acquired EGFR-TKI resistance and had undergone repeat biopsies. A total of 240 patients (50.21%) presented the T790 M mutation. Among the 141 patients who were tested for MET amplification, 14 (9.93%) tested positive. Eight (6.20%) of the 129 patients with NSCLC exhibited histological transformation to SCLC. In addition, some patients presented with rare causes of drug resistance, including PIK3CA, HGF overexpression and HER2 amplification. However, the incidence of these cases was too low to conduct an independent analysis (Fig. 2).

There was no significant difference in gender, age, or smoking habits between T790 M-positive and T790 M-negative patients. However, OS was significantly greater in T790 M-positive patients compared with T790 M-negative patients (Table 2).

**Table 2:** Clinical Characteristics

	Gender(n)		Age(n)		Smoking status(n)		OS* (months)
	Man	Female	<70	≥70	Smoker	Non-smoker	
T790 M(+)	73	133	84	22	46	136	41.6
T790 M(−)	81	125	102	27	45	127	30.3

*P < 0.05 (data from the research11, 12, 13, 16 and19; *n* = 309)

**Fig. 3:**
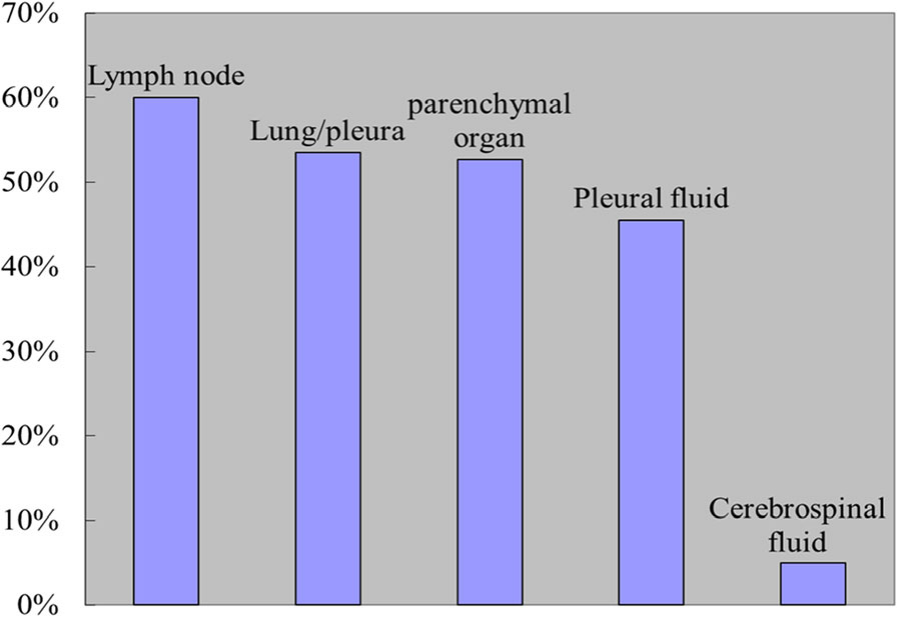
The positive rate of T790 M at different biopsy parts

The T790 M mutation was detected in 21 (60.0%) of the 35 repeat lymph node biopsy specimens and in 129 (53.5%) of the 241 lung and pleural biopsy specimens. As the number of single solid organ biopsy samples (including the skin, liver, brain, adrenal gland and bone) was small, it was difficult to conduct an independent analysis of these samples. The overall rate of T790 M mutation in the solid organ biopsies was 52.7% (39/74), similar to that observed in lung and pleural biopsy samples. The rate in pleural effusion specimens was 45.5% (15/33). The T790 M mutation was detected in less than 5% of cerebrospinal fluid specimens (1/21), which was significantly lower than that in other types of specimens (*P* < 0.05) (Fig. 3).

**Fig. 4:**
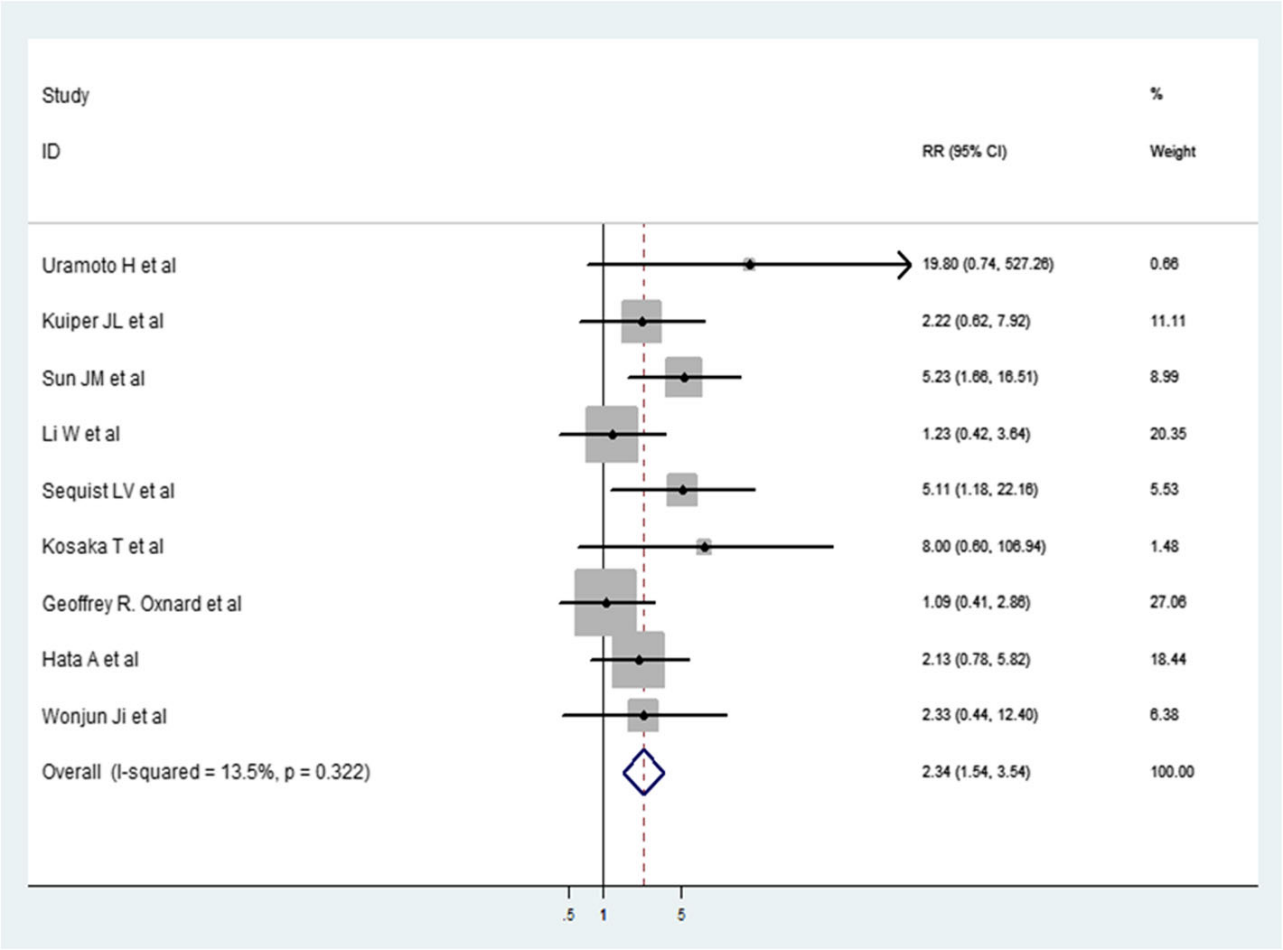
Meta-analysis about positive ratio of acquired T790 M mutation in patients with exon19 deletion and L858R point mutation. (One study [18] cannot be included due to not providing the type of mutation with initial biopsy)

In 240 T790 M-positive NSCLC patients, 27 patients had secondary biopsy results inconsistent with the initial results; as this may be related to the temporal heterogeneity of the organization and spatial heterogeneity, these findings were not included in the comparison. The remaining 213 patients had sensitive mutations (55 patients with the exon 21 L858R point mutation and 158 patients with the del19 mutation). The 55 T790 M-positive patients with the L858R mutation accounted for 36.42% of total patients with L858R, and the 158 T790 M-positive patients with the del19 mutation accounted for 58.30% of total patients with del19 (36.42% vs 58.30%, *P* < 0.01). The meta-analysis also revealed that the prevalence of the T790 M mutation was significantly greater in patients with the del19 mutation compared with the L858R mutation (HR: 2.34, 95% CI: 1.54–3.54, *P* < 0.05, Begg’s Test Pr > ∣Z∣ = 0.048) (Fig. 4).

## Discussion

D Ross et al. reviewed the acquired resistance to EGFR TKI in lung cancer and found that the second EGFR mutation comprised approximately 60% (the T790 M mutation was approximately 40–55%), the bypass activation mutation was approximately 20% (Met amplification 5%, HER2 amplification 8–13%, Sclc 10%, BRAF 1%, PIk3CA 1–2% and EMT 1–2%), and other unknown mechanisms accounted for 15–20% [21]. The incidence of T790 M mutations in patients with NSCLC was between 33% and 60% in the previous study, and these differences were predominantly attributable to differences in the detection method and sample size. The primary cause of acquired resistance to TKIs in patients with NSCLC is the T790 M mutation, followed by MET amplification and SCLC transformation. The 10 studies included in the present meta-analysis used different methods, which might have had differing sensitivities for detecting the T790 M mutation. However, as all studies used common clinical testing methods, the observed incidence of T790 M (50.21%) might be clinically relevant. Several new agents that target T790 M, including AZD9291 and HM61713, are being investigated in phase III trials of NSCLC patients with EGFR mutations and T790 M-mediated TKI resistance. These agents have shown extremely promising results, and AZD9291 has been approved by the US Food and Drug Administration [22, 23]. Given the complexity of the various mechanisms underlying EGFR-TKI resistance, repeat biopsies are required to clarify the precise mechanism underlying this phenomenon in patients with NSCLC.

Patients with the T790 M mutation, irrespective of EGFR activating mutation subtypes, had better survival beyond EGFR-TKI progression, which suggested that resistance acquired through the T790 M mutation might follow a more indolent course than clinical resistance without the mutation. Several clinical studies [16, 17, 24] reported similar results in Caucasian population and East-Asia population, respectively. Notably, preclinical data have also demonstrated that acquisition of the T790 M mutation is associated with more indolent growth than in parental cell lines without T790 M mutation in the absence of TKI selection [25]. The results have validated previous findings and further elucidated the association between T790 M-independent mechanisms and the better survival of patients with del19.

Patients with del19 treated with first-generation TKIs showed a greater clinical benefit than that of patients with L858R mutations [24, 26]. Yang et al. reported differences in prognosis among NSCLS patients with different sensitive mutations who underwent afatinib treatment. Drug resistance is the most important factor affecting the disease process, and the T790 M mutation is the most common drug resistance mechanism. To eliminate the limitation of inadequate sample size and confirm these findings, we conducted a systematic review of studies published over the past 10 years and found that patients with del19 had a higher incidence of T790 M mutation compared to patients with L858R (58.30% vs 36.42%).

Exon 19 deletion mutations are more likely to develop T790 M mutations, and T790 M mutant cell lines proliferate slowly [26], which may be one of the possible mechanisms for the improved prognosis of patients with del19 after TKI treatment. T790 M mutation patients are more likely to receive effective follow-up treatment, which may also extend the OS of NSCLC patients with del19.

We also analyzed the association of the T790 M mutation with the clinicopathological features of NSCLC patients. The prevalence of the T790 M mutation varied according to the sites of the repeat biopsy, with the highest rate observed in metastatic lymph nodes biopsies (60%), followed by lung and pleural biopsies (53.5%), biopsies from other parenchymatous organs (52.7%) and pleural effusion biopsies (45.5%). Although these differences were not statistically significant, they deserve attention because pleural effusion is one of the most common biopsy sites in patients with NSCLC. In addition, the observation that OS was increased in T790 M-positive patients indicates that T790 M might be a useful marker for predicting the prognosis of NSCLC patients who underwent TKI therapy.

Of note, there are several limitations in our study. First, none of the studies included in the analysis were randomized controlled studies, the number of studies was small, and some sample size were too small, those might cause possible result publication bias. Second, although the T790 M mutation accounts for 50% of cases of acquired TKI resistance, the other mechanisms mediating acquired resistance were not examined in this study. Third, unfortunately, we did not include one study that evaluated a large number of repeat biopsies [9] because the authors did not report the type of sensitizing mutations in patients with NSCLC with the T790 M mutation. Therefore, we had to include another study from the same research center [16].

As the T790 M mutation is associated with improvements in OS in patients with NSCLC, it might serve as a prognostic factor. The higher incidence of the T790 M mutation in patients with del19 compared with exon 21 L858R might account for the higher OS rate observed in patients with del19; however, this hypothesis deserves further investigation.

## Conclusions

The T790 M mutation is the primary cause of EGFR-TKI resistance. Considering the survival benefit of the T790 M mutation and the finding that T790 M occurred more frequently in patients with del19 than those with exon 21 L858R, this may explain why the patients with del19 had an improved OS.

## Abbreviations

CI: Confidence interval
DDPCR: Droplet Digital Polymerase Chain Reaction
EGFR: Epidermal growth factor receptor
HR: Hazard ratio
NSCLC: Non-small cell lung cancer
OS: Overall survival
PFS: Progression-free survival
T790 M: The substitution of threonine with methionine at amino acid position 790
TKIs: Tyrosine kinase inhibitors
